# Acute effects of exercise-induced muscle damage on sprint and change of direction performance: A systematic review and meta-analysis

**DOI:** 10.5114/biolsport.2024.131823

**Published:** 2024-01-30

**Authors:** Drew C. Harrison, Kenji Doma, Catherine Rush, Jonathan D. Connor

**Affiliations:** 1 James Cook University, College of Healthcare Sciences, Sports and Exercise Science, Australia; 2 James Cook University, Biomedical Sciences

**Keywords:** Athletic Performance, Creatine Kinase/blood, Myalgia, Plyometric Exercise, Resistance Training

## Abstract

The aim of this study is to determine the acute effects of resistance and plyometric training on sprint and change of direction (COD) performance in healthy adults and adolescents. A systematic literature search was conducted via Medline, Cinahl, Scopus and SportDiscus databases for studies that investigated: 1) healthy male, female adults, or adolescents; and 2) measured sprint or change of direction performance following resistance and plyometric exercises. Studies were excluded if: 1) resistance or plyometric exercises was not used to induce muscle damage; 2) conducted in animals, infants, elderly; 3) sprint performance and/or agility performance was not measured 24 h post muscle damaging protocol. Study appraisal was completed using the Kmet Quality Scoring for Quantitative Study tool. Forest plots were generated to quantitatively analyse data and report study statistics for statistical significance and heterogeneity. The included studies (*n* = 20) revealed sprint and COD performance was significantly impaired up to 72 hr following resistance and plyometric exercises; both protocols significantly increased creatine kinase (CK), delayed-onset muscle soreness (DOMS) and decreased countermovement jump (CMJ) up to 72 hr. The systematic review of 20 studies indicated that resistance and plyometric training significantly impaired sprint and COD performance up to 72 hours post-exercise. Both training protocols elevated exercise-induced muscle damage (EIMD) markers (CK, DOMS) and decreased CMJ performance within the same timeframe.

## INTRODUCTION

The health benefits of physical exercise are irrefutable [1, 2]. However, strenuous exercise was shown to cause immediate and delayed alterations in cardiovascular function [3, 4]. Furthermore, strenuous exercise may also result in high levels of exercise-induced muscle damage (EIMD), particularly for untrained people [5]. EIMD symptoms can occur due to unfamiliar eccentric contractions which are known to cause significant structural damage [5]. The resulting structural damage is commonly measured by outcomes that encapsulate EIMD, such as creatine kinase (CK), delayed-onset muscle soreness (DOMS), and muscular contractility [6–13]. Blood biomarkers, such as CK, can be used as indicators of muscle damage [14]. It has been speculated that the disruption of the intermediate filaments reportedly activates Groups III and IV afferents, which cause an individual to experience DOMS [15]. Despite the clear impairment in muscle function, classical studies since the 1980’s focused on muscle function using mono-articular isometric or isokinetic contractions, which may not be applicable to sporting populations.

Incorporating ‘multi-articular’ activities as both EIMD protocols and motor performance measures in EIMD research would improve the application of findings to real-world practice in sports. Multi-articular exercises commonly practiced by athletes known to cause EIMD include resistance (squats, bench press, latpull down) [16-19], and plyometric exercises (various jump protocols, explosvie push-ups) [20-24]. To date, several studies [18, 20, 22–25] have reported impairment in both sprint and COD performance for several days post-exercise with a concomitant increase in CK and DOMS. Conversely, numerous studies have reported no differences in sprint [19, 21, 26–29] and COD [16, 21, 22] performance following a bout of resistance or plyometric training. The discrepancy between these findings could be attributed to variations in exercise prescription (i.e., resistance or plyometric), the type of sprint and COD protocols, athletes’ training histories and skill level of the participants between studies. These factors are known to influence the effects of EIMD [30], thus a systematic review evaluating the variation in study design will provide a comprehensive overview of prior research as well as highlight the methodological limitations to improve the quality of future research.

Since 2018, several systematic reviews have investigated the impact of EIMD on various performance measures. For example, a series of meta-analyses conducted by Doma and colleagues [31–33] demonstrated that EIMD impaired vertical jump, isometric contractions, and isokinetic contractions, with concomitant increases in CK and DOMS for up to 48 hours after a variety of muscle-damaging protocols, including isokinetic eccentric contractions and downhill running. Whilst the meta-analyses by Doma et al [31–33] clearly shows that EIMD impaired muscle function for up to 48 hours, the studies included involved a variety of muscle-damaging protocols (e.g., isokinetic contractions and downhill running) and muscle performance measures (isokinetic and isometric contractions) that does not reflect real-world training practice. Silva et al [34] conducted a systematic review and meta-analysis and reported that vertical jump and sprint performance was impaired following competitive and simulated soccer matches. Whilst the use of soccer matches as a muscle-damaging protocol may replicate real-world exercise conditions, workloads may vary between positions, and the intensity is unable to be controlled due to the different team-specific tactics. Furthermore, a meta-analysis which concentrates on exercises widely used for athletic development, such as resistance and plyometric training, which assess their acute effects on performance outcomes observed in sport, like sprint and COD, would be of value. Thus, the purpose of this systematic review and meta-analysis was to determine the acute effects of resistance and plyometric training on sprint and COD performance in healthy adults or adolescents. We hypothesise that both muscle damaging protocols will significantly impair sprint and COD performance for up to 48 hours post-exercise.

## MATERIALS AND METHODS

The current systematic review was conducted in accordance with the PRISMA guidelines [35] and followed a population, intervention/ exposure, comparison, and outcome (PICO) approach.

1. *Population:* healthy adults or adolescents.
2. *Intervention:* resistance or plyometric exercises.
3. *Comparison:* the outcome measures were compared at 24-, 48-, and 72-hours after the muscle damaging protocols.
4. *Outcome:* the outcome measures included fundamental movement patterns (i.e., sprint and COD), muscle damage biomarkers (i.e., creatine kinase), and subjective measures of muscle soreness (i.e., visual analogue scale).

Studies were excluded if: (1) they did not use resistance or plyometric exercises to induce muscle damage; (2) were conducted in animals, infants, elderly; (3) outcome measures were examined for chronic adaptation; (4) outcome measures were not compared 24 h post muscle damaging protocol; (5) sprint performance and/or agility performance was not measured; (6) they were published in non-English language; (7) results were published as a conference abstract, review, or case report.

### Search strategy

A search of scientific literature was conducted on October 10^th^, 2022, via four major electronic databases (Medline, Cinahl, Scopus and SportDiscus). MeSH terms and free text searches were combined. The subject headings were categorised into three strings, including: (1) humans; (2) motor performance (sprint or jump or COD); (3) biomarkers of muscle damage (Creatine Kinase/blood OR Myalgia). The following free text search terms were used: (eccentric or resistance training or exercise) and (“muscle damage” or “creatine kinase” or soreness).

### Selection process

Two authors (JC and DH) independently screened and processed the literature search. First, all abstracts were highlighted as either ‘green’ (definitively meeting the criteria), ‘yellow’ (possibly meeting the criteria) or ‘red’ (not meeting the criteria). After independent screening, the inter-rater reliability of the two reviewer’s inclusions was assessed using 40% of the screened abstracts. A Weighted Kappa statistic of 0.78 revealed the inter-rater reliability was acceptable (95% confidence interval: 0.69-0.88) [36]. Full text was then extracted from the selected articles and subjected to further screening for additional relevant publications in accordance with the inclusion/exclusion criteria.

### Data extraction, assessment of quality and risk of bias

An excel spreadsheet was used to extract descriptive information involving (1) study; (2) research design (i.e., randomised, placebo control or cross-over); (3) sample size; (4) physical characteristics; (5) training background; (6) EIMD protocol; (7) exercise type; (8) outcome measures; and (9) post-exercise time points of when outcome measures were collected (i.e., 24-, 48-, 72-hours after the muscle damaging protocol). Means ± standard deviations from pre- and post- exercise outcome measures were extracted in preparation for the current meta-analyses. Any outcome measures reported as standard error or confidence intervals, or median quartiles were converted using appropriate methods [37]. Each study was critically appraised using a modified Kmet rating scale [38]. The Kmet quality ratings were classified as either excellent (1.00–0.8), good (0.79–0.60), fair (0.59–0.50), and poor (0–0.049). Additional criteria were incorporated to the original 14-point scoring system to assess methodological characteristics of each study, including: (1) adequate description of EIMD protocol (i.e., load, volume, rest); (2) sufficient detail of participants’ training history; (3) participants being instructed to abstain from supplementation or recovery protocols. Studies were given a score of “2” if the specific criteria were met, a “1” if partially met and “0” if not met. Studies with non-applicable items were denoted with “n/a” and excluded from the final score. Two authors completed the critical appraisal of each study independently to establish an inter-rater reliability of the Kmet scoring procedure.

### Statistical analysis

A meta-analysis was conducted using RevMan. To account for interstudy heterogeneity, forest plots were generated via a random effects model. All data points from post-exercise time points (i.e., 24–72-hours post muscle-damaging protocol) were collected to calculate the magnitude of differences when compared to baseline values. Small, medium, and large (0.2, 0.5, 0.8) were values used to denote magnitude of change based upon the standardised mean differences (SMD). The level of statistical significance of the repeated measures was determined from p-values (0.05). Differences in study design was controlled using separate equations to estimate effect of SMD and standard errors [39].

## RESULTS

### Systematic literature search

**FIG 1:**
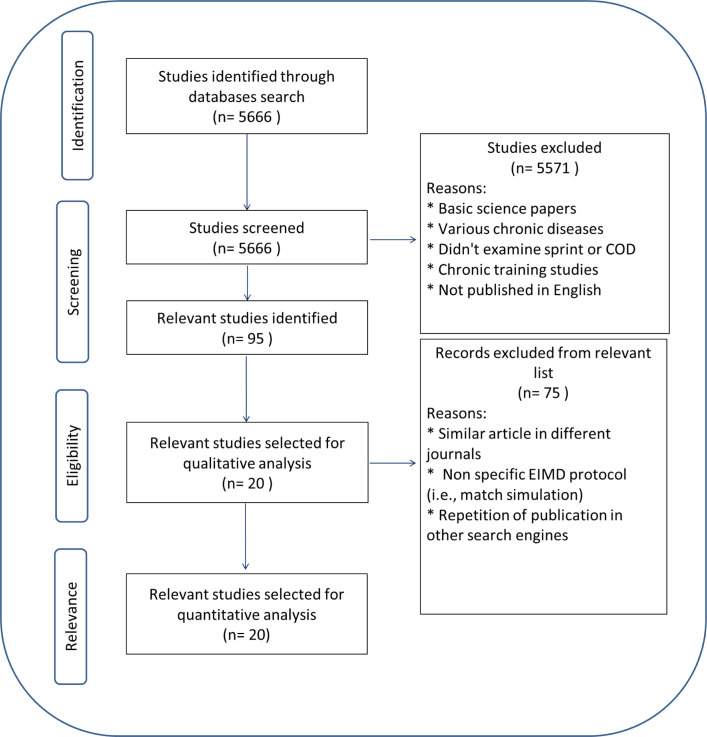
Schematic of the PRISMA flowchart.

The scientific literature search of Medline, Cinahl, Scopus and Sport- Discus databases yielded 9232 abstracts. Duplicates were removed (n = 3565) and a total of 5667 abstracts were screened according to the inclusion criteria (Fig 1). After screening was completed, another 5647 abstracts were excluded, leaving 20 full text articles for inclusion (Table 2).

### Participants

Table 1 displays participant physical characteristics, sample size and outcome distribution at baseline for the placebo (PLA) conditions for each study. There was a total of 208 participants which had a collective mean ± standard deviation for age, height, and body mass of 21.4 ± 2.6 years, 1.78 ± 0.8 m, and 75.3 ± 7.8 kg.

**TABLE 1:** Participant characteristics and baseline comparisons of outcome measures

Study	Sample Size	Physical Characteristics	Training background
AkdenİZ et al. [20]	n = 11	Age 21.6 ± 1.9 years; height 1.76 ± 0.05 m; body mass 70.4 ± 3.7 kg; BMI 23.5 ± 3.6 kg/m^2^	Male, Detrained

Cockburn et al. [40]	EXP: n = 7 CON: n = 7	Age 24 ± 4 years; height 1.83 ± 0.07 m; body mass 70.4 ± 3.7 kg	Male, Trained

Cook and Beaven [41]	n = 12	Age 23.3 ± 1.4 years; height 1.85 ± 0.04 m; body mass 96.7 ± 10.8 kg	Male, RT trained

de Freitas et al. [16]	EXP: n = 6 CON: n = 6	Age 24.8 ± 4.7 years; height 1.94 ± 0.07 m; body mass 92.5 ± 9.8 kg	Male, RT trained

de Oliveira et al. [21]	EXP: n = 11 CON: n = 10	Age 17 ± 0.3 years; height 1.77 m; body mass 67.1 ± 2.3 kg	Male, RT trained

Doma et al. [22]	n = 9	Age 25 ± 5 years; height 1.77 ± 0.06 m; body mass 75 ± 8 kg	Female (1) Male (8), RT untrained

Doma et al. [18]	n = 8	Age 15 ± 1.7 years; height 1.80 ± 0.1 m; body mass 67.9 ± 7.9 kg	Male, RT untrained

Doma et al. [17]	n = 16	Age 21 ± 1 years; height 1.71 ± 0.05 m; body kg mass 77.8 ± 12.6	Female, RT trained

French et al. [42]	EXP: n = 20 CON: n = 6	Age 21.5 ± 2 years; height 1.87 ± 0.08 m; body mass 79.4 ± 12.1 kg	Male, RT trained

Getto and Golden [26]	EXP: n = 15 CON: n = 8	M = Age not stated; height 1.88 ± 0.1 m; body mass 113.2 ± 20.2 kg F = Age not stated; height 1.77 ± 0.11 m; body mass 74.1 ± 14.3 kg	Female (10) Male (13), RT trained

Goulart et al. [27]	n = 10	Age 22.2 ± 5.3 years; height 1.65 ± 0.06 m; body mass 59.8 ± 6.1 kg	Female, RT trained

Harrison et al. [19]	n = 10	Age 18 ± 2 years; height 1.78 ± 5.3 m; body mass 71.2 ± 4.2 kg; BMI 23.2 ± 5.0 kg/m^2^	Male, RT untrained

Highton et al. [23]	EXP: n = 7 CON: n = 5	Age 21 ± 1.2 years; height 1.77 ± 0.11 m; body mass 70.8 ± 8.2 kg	Not stated, RT untrained

Kargarfard et al. [25]	EXP: n = 15 CON: n = 15	Age 28.1 ± 3.3 years; height 1.74 ± 0.08 m; body mass 79.3 ± 10.8 kg; BMI 26.2 ± 1.9 kg/m^2^	Male, RT trained

Rankin et al. [43]	n = 18	Age 21.6 ± 3.4 years; height 1.62 ± 0.09 m; body mass 64.3 ± 6.1 kg	Female, Trained

Santos-Mariano et al. [28]	n = 11	Age 18.7 ± 2.7 years; height 1.81 ± 0.08 m; body mass 69.9 ± 6.4 kg	Male, RT Trained

Semark et al. [29]	EXP: n = 13 CON: n = 12	Age 19 ± 3 years; height 1.77 ± 8 m; body mass 70 ± 8 kg	Male, Trained

Twist and Eston [24]	n = 10	Age 22.4 ± 3.2 years; height 1.79 ± 0.05 m; body mass 80.6 ± 10.7 kg	Male, Trained

VanHeest et al. [44]	EXP: n = 24 CON: n = 12	Age 20 ± 0.01 years; height 1.78 ± 0.03 m; body mass 78.7 ± 3.9 kg	Male, Trained

Zinner et al. [45]	n = 12	Age 22 ± 4years; height 1.87 ± 0.07 m; body mass 89.7 ± 12.7kg	Not specified, Trained

M – male; F – female.

**TABLE 2:** Methodological description for muscle damaging protocol, type of outcome measure and study timeline.

Study	EIMD Protocol	Fundamental Biomarker DOMS	Time Points
AkdenİZ et al. [20]	Drop jumps 5 sets × 20 repetitions, 60 cm, 2 minutes rest between sets and 10 second intervals between jumps	30 m sprint and Illinois agility None VAS 0–10	Baseline, 1-hr, 24-hr, 48-hr, 72-hr, 96-hr

Cockburn et al. [40]	Unilateral eccentric-concentric knee flexions 6 sets × 10 repetitions at 1.05 rad∙s-1	15 m sprint, T-test agility, CMJ and DJ CK VAS 0–10	Baseline, 24 hr, 48 hr, 72 hr post

Cook and Beaven [41]	Gym and track-based conditioning session 60 minute, high intensity	40 m RSA None None	24 hr post

de Freitas et al. [16]	Bench press, incline bench press, decline bench press, barbell shoulder press, leg press, dumbbell squat; leg extension machine, cable pull down—pro lat bar, wide grip seated row, dumbbell bent over row, barbell curl, leg press, seated leg curl and seated hip adduction machine, 3 3 × 8–10 maximal repetitions, with 1.5–2 minutes recovery.	Square agility, SJ, CMJ CK VAS 0–10	Baseline, 24 hr post

de Oliveira et al. [21]	Box jumps 5 sets × 20 repetitions, 60 cm, 2 minutes rest between sets. Squatting leg extensions and leg curls 4 sets of 8 repetition maximum (RM), 2 minutes rest between sets.	35 m RSA, T-test agility and CMJ CK VAS 0–10	Baseline, 24 hr, 48 hr, 72 hr post

Doma et al. [22]	Split squat jumps 3 sets × 12 repetitions, 1.5 minutes rest between sets. Weighted squat jumps 3 sets × 7 repetitions with 35% body mass (BM), 2 minutes rest between sets. Depth jumps 3 sets × 7 repetitions, 1.5 minutes rest between sets. Tuck jumps 3 sets × 12 repetitions, 2 minutes rest between sets. Walking lunges 3 sets × 12 repetitions with 35% BM, 3 minutes rest between sets. Single-leg dumbbell Romanian deadlifts 3 sets × 15 repetitions with 50% BM, 2 minutes rest between sets. Sit-ups 3 sets × AMRAP with 10% BM, 2 minutes rest between sets.	20 m sprint and DJ CK VAS 0–10	Baseline, 24 hr, 48 hr

Doma et al. [18]	Incline leg press, chest press, single-leg horizontal leg press, seated row and triceps extensions 3 sets × 10 repetitions each exercise at 70% of 1 or 6 RM, 1.5 minutes rest between sets. Walking lunges 3 sets × 20 m with 30% BM, 1.5 minute between sets.	15 m sprint, CMJ and DJ CK VAS 1–10	Baseline, 24 hr, 48 hr post

Doma et al. [17]	Power cleans 3 sets × 5 repetitions at 80% 1RM. Deadlift 5 sets × 5 repetitions at 80% 1 RM. Dumbbell shoulder press 3 sets × 10 repetitions at 70% 1RM. Back squats 5 sets of 5 repetitions at 80% 1RM. Dumbbell lunges 4 sets × 10 repetitions at 40% BM. 2 minutes rest between each set and exercise.	T-test agility and CMJ None Vas 1–10	Baseline, 24 hr, 48 hr post

French et al. [42]	Squats 6 sets × 10 repetitions at 100% BM	10, 30 m sprint, Multiplanar agility and CMJ CK VAS 100 mm	Baseline, 1 hr, 24 hr, 48 hr

Getto and Golden [26]	Sprints 8 reps × 70 yrd and various plyometric exercises	20 m sprint and CMJ None VAS 1–10	Baseline, 24 hr post

Goulart et al. [27]	Half-squat, jump squat, deadlift and lunge 3 sets of 6 repetitions at 50% 1RM	20 m sprint and CMJ None VAS 0–10	Baseline, 24 hr, 48 hr post

Harrison et al. [19]	Incline leg press, chest press, single-leg horizontal leg press, seated row and triceps extensions 3 sets × 10 repetitions each exercise at 70% of 1 or 6 RM, 2 minutes rest between sets. Walking lunges 3 sets × 20 m with 10–30% BM, 2 minutes between sets.	15 m sprint, CMJ CK VAS 1–10	Baseline, 24 hr, 48 hr post

Highton et al. [23]	Maximal vertical jumps 10 sets × 10 repetitions, 1 minute rest between sets.	10 m sprint, 505–agility and CMJ None VAS 1–10	Baseline, 24 hr, 48 hr, 168 hr post

Kargarfard et al. [25]	Squats and leg press AMRAP at 75–77% 1RM	T-test agility and CMJ CK VAS 100 mm	Baseline, 24 hr, 48 hr, 72 hr post

Rankin et al. [43]	Sprints with decelerations and plyometric jumps 8 sets × 10 repetitions	20 m sprint, CMJ and DJ CK VAS 0–10	Baseline, 24 hr, 48 hr, 72 hr

Santos-Mariano et al. [28]	Eccentric half-squat 4 sets × 12 repetitions at 70% 1RM, 2 minutes rest between each set	30 m sprint and CMJ CK VAS 0–10	Baseline, 24 hr, 48 hr, 72 hr post

Semark et al. [29]	Drop jumps 7 sets × 10 repetitions, 50 cm, 1 minute rest between sets	30 m sprint CK VAS 0–10	Baseline, 12 hr, 24 hr, 48 hr, 72 hr

Twist and Eston [24]	Maximal vertical jumps 10 sets × 10 repetitions, 1 minute rest between sets.	10 m sprint CK VAS 0–10	Baseline, 0.5 hr, 24 hr, 48 hr, 72 hr post

VanHeest et al. [44]	Seated knee flexion 100 repetitions at 120% 1RM	T-test agility CK VAS 100 mm	24 hr, 48 hr, 72 hr, 96 hr, 120 hr post

Zinner et al. [45]	Sprints 30 repetitions × 30 m and CMJ 2 repetitions	30 m RSA and CMJ CK Likert-type 0–6	Baseline, 48 hr post

VAS- Visual analog scale; CK- creatine kinase; RM- repetition maximum; AMRAP – as many repetitions as possible

### Methodological descriptions

The most frequently utilized sprint performance protocol was 30 m sprint (4 studies), followed by 20 m sprint (3 studies), 15 m sprint (3 studies), 10 m sprint (2 studies), 40 m, 50 m, 100 m sprint with one study each. Only two studies investigated repeated sprint ability over 10 m and 35 m distances, respectively. The most common agility performance protocol was the T-test agility (5 studies), followed by the Illinois agility test (2 studies), Square test (1 study), Y-test (1 study) and M-test (1 study). The most reported biomarker of muscle damage was CK (12 studies), whilst only two studies reported myoglobin and six studies reported no blood biomarkers. CMJ was the most reported jump performance measure (14 studies) whilst only four studies assessed drop jump (DJ) performance. The most reported DOMS measure was VAS 0–10 (13 studies), other measures included 5-point Likert scale (1 study), 10-point Likert scale (1 study), Total quality recovery (2 studies), 10 cm pain scale (2 studies), Navigate pain application (1 study) and a 6-point acute recovery and stress scale (ARSS).

### Methodological quality

The Kmet scaling scores ranged from 0 to 2 quality (Table 3.). All included studies met the following criteria: appropriate objective and study design; participant characteristics and pre-exercise variables; and defined performance outcome measures. The Kmet items that were reported least included: resistance training history; control of factors that may bias the results (i.e., supplements, recovery interventions, familiarisation); and sample size appropriation.

**TABLE 3:** Kmet scores of all included studies

Study	1	2	3	4	5	6	7	8	9	10	11	12	13	14	Ratings	Quality
AkdenİZ et al. [20]	2	2	2	2	2	1	2	2	2	2	2	2	2	2	27/28	Excellent
Cockburn et al. [40]	2	2	2	2	2	1	2	2	1	1	2	2	2	1	24/28	Excellent
Cook and Beaven [41]	2	2	2	2	0	2	2	2	1	1	2	0	2	1	21/28	Good
de Freitas et al. [16]	2	2	2	2	2	2	2	2	2	2	2	1	2	2	27/28	Excellent
de Oliveira et al. [21]	2	2	2	2	2	2	2	2	2	2	2	2	2	2	28/28	Excellent
Doma et al. [22]	2	2	2	2	2	2	2	2	2	2	2	2	2	2	28/28	Excellent
Doma et al. [17]	2	2	2	2	2	2	1	2	2	2	2	2	2	2	27/28	Excellent
Doma et al. [18]	2	2	2	2	2	2	0	2	1	2	2	2	2	2	25/28	Excellent
French et al. [42]	2	2	1	2	2	2	2	2	2	2	2	2	2	2	27/28	Excellent
Getto and Golden [26]	2	2	2	2	2	1	1	2	1	2	2	1	2	2	24/28	Excellent
Goulart et al. [27]	2	2	2	2	2	2	1	2	1	2	2	2	2	2	26/28	Excellent
Harrison et al. [19]	2	2	1	2	2	2	2	2	2	2	2	2	2	2	27/28	Excellent
Highton et al. [23]	2	2	2	2	2	2	0	2	2	2	2	1	2	2	25/28	Excellent
Kargarfard et al. [25]	2	2	2	2	2	2	2	2	1	2	2	1	2	1	25/28	Excellent
Rankin et al. [43]	2	2	2	2	2	0	2	2	2	1	2	2	2	2	25/28	Excellent
Santos-Mariano et al. [28]	2	2	2	2	2	2	1	2	2	2	2	2	2	2	27/28	Excellent
Semark et al. [29]	2	2	1	2	2	1	1	2	2	2	2	2	2	2	25/28	Excellent
Twist and Eston [24]	2	2	1	2	2	1	2	2	2	2	2	2	2	2	26/28	Excellent
VanHeest et al. [44]	2	2	1	2	2	2	1	2	0	2	2	2	0	0	20/28	Good
Zinner et al. [45]	2	2	2	2	2	1	2	2	2	2	1	2	2	1	25/28	Excellent

### Quantitative analyses

The meta-analysis revealed that EIMD protocols significantly decreased sprint performance at 24-hours (Z = 13.71; p = ≤ 0.01; SMD = 1.43; Figure 2a), 48-hours (Z = 12.19; p = ≤ 0.01; SMD = 1.37; Figure 2b) and 72-hours (Z = 10.15; p = ≤ 0.01; SMD = 1.22; Figure 2c) post-exercise, with low-to-high interstudy heterogeneity (I^2^ = 0%, 26% and 0%, respectively). The COD performance measures were also significantly decreased by EIMD at 24-hours (Z = 8.37; p = ≤ 0.01; SMD = 1.17; Figure 3a), 48-hours (Z = 6.98; p = ≤ 0.01; SMD = 1.48; Figure 3b) and 72-hours (Z = 7.70; p = ≤ 0.01; SMD = 1.01; Figure 3c) post-exercise, with low-to-moderate interstudy heterogeneity (I^2^ = 30%, 61% and 0%, respectively). The EIMD protocols also significantly increased CK, at 24-hours (Z = 7.52; p = ≤ 0.01; SMD = 2.16; Figure 4a), 48-hours (Z = 7.06; p = ≤ 0.01; SMD = 2.12; Figure 4b) and 72-hours (Z = 4.32; p = ≤ 0.01; SMD = 1.91; Figure 4c) post-exercise, with moderate-to- high inter-study heterogeneity (I^2^ = 66%, 67% and 76%, respectively). For DOMS, perceived measures were significantly increased at 24-hours (Z = 7.28; p = ≤ 0.01; SMD = 3.07; Figure 5a), 48-hours (Z = 6.89; p = ≤ 0.01; SMD = 3.29; Figure 5b) and 72-hours (Z = 5.06; p = ≤ 0.01; SMD = 3.29; Figure 5c) post-exercise, with moderateto-high interstudy heterogeneity (I^2^ = 77%, 78%, 65%, respectively). Finally, CMJ measures were significantly decreased at 24-hours (Z = 11.53; p = ≤ 0.01; SMD = 1.20; Figure 6a), 48-hours (Z = 12.19; p = ≤ 0.01; SMD = 1.24; Figure 6b) and 72-hours (Z = 8.59; p = ≤ 0.01; SMD = 1.26; Figure 6c) post-exercise, with low interstudy heterogeneity (I^2^ = 0%).

**FIG. 2a:**
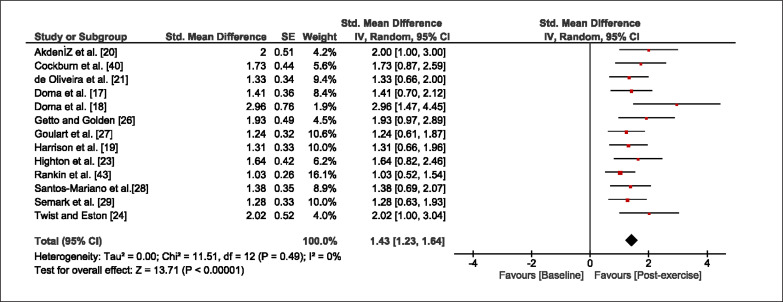
Forest plot for sprint performance at 24-hours after the muscle damaging protocol

**FIG. 2b:**
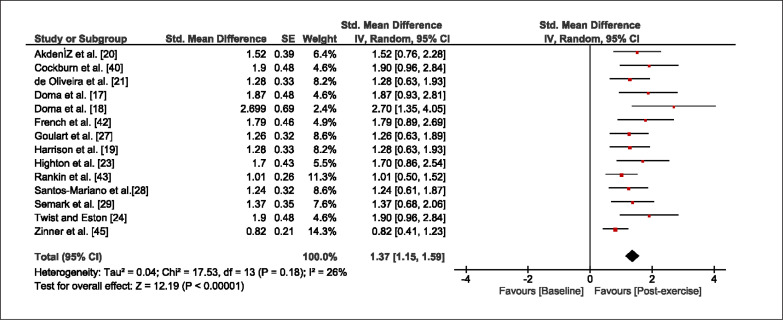
Forest plot for sprint performance at 48-hours after the muscle damaging protocol

**FIG. 2c:**
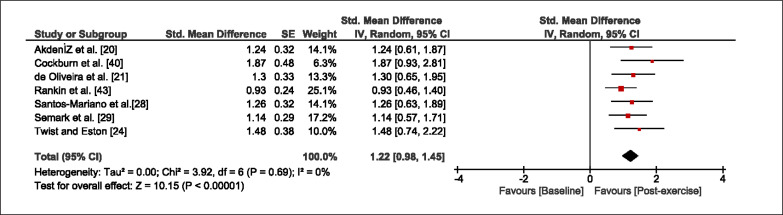
Forest plot for sprint performance at 72-hours after the muscle damaging protocol

**FIG. 3a:**
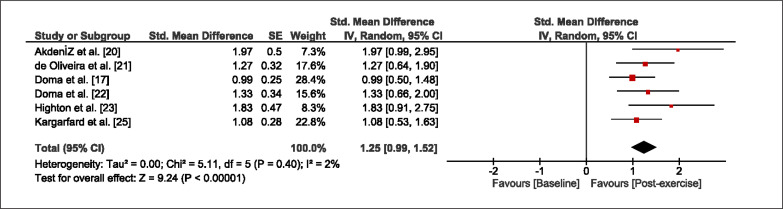
Forest plot for COD performance at 24-hours after the muscle damaging protocol

**FIG. 3b:**
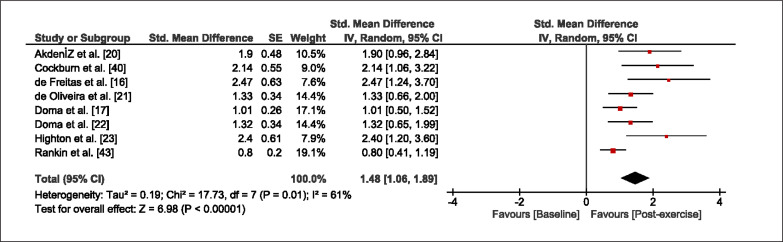
Forest plot for COD performance at 48-hours after the muscle damaging protocol

**FIG. 3c:**
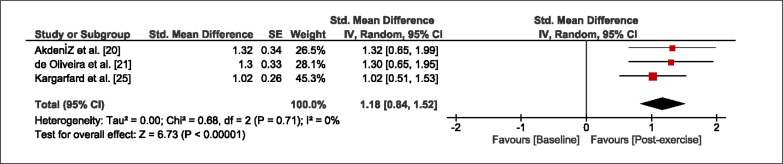
Forest plot for COD performance at 72-hours after the muscle damaging protocol

**FIG. 4a:**
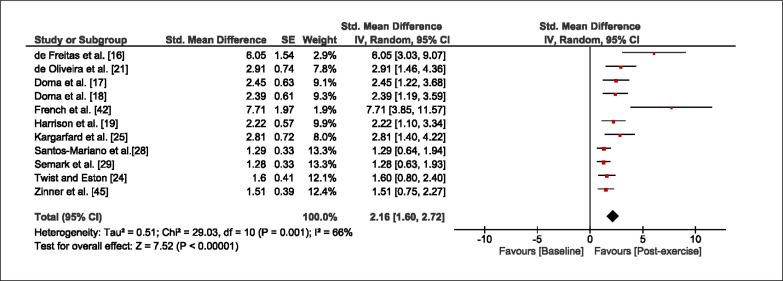
Forest plot for CK at 24-hours after the muscle damaging protocol

**FIG. 4b:**
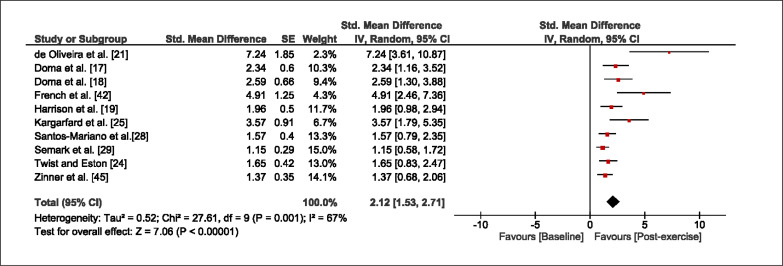
Forest plot for CK at 48-hours after the muscle damaging protocol

**FIG. 4c:**
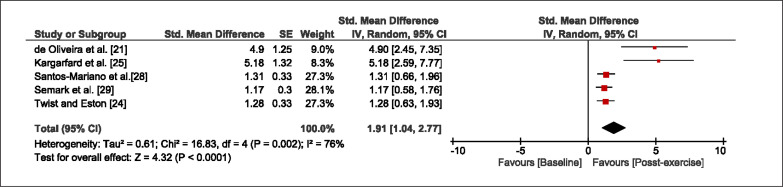
Forest plot for CK at 72-hours after the muscle damaging protocol

**FIG. 5a:**
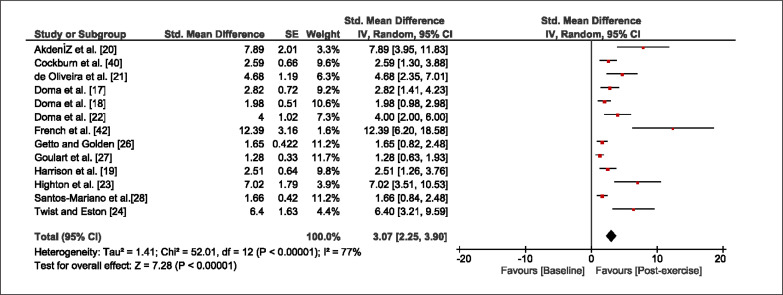
Forest plot for perceived muscle soreness at 24-hours after the muscle damaging protocol

**FIG. 5b:**
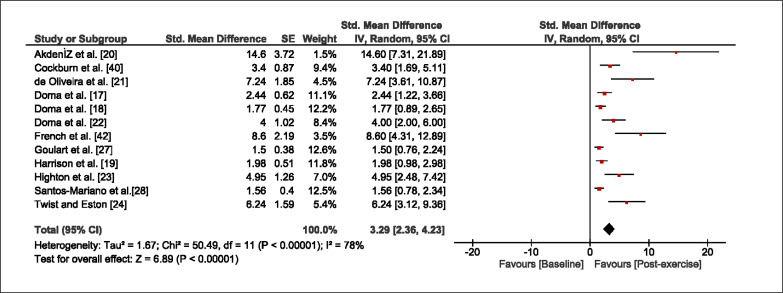
Forest plot for perceived muscle soreness at 48-hours after the muscle damaging protocol

**FIG. 5c:**
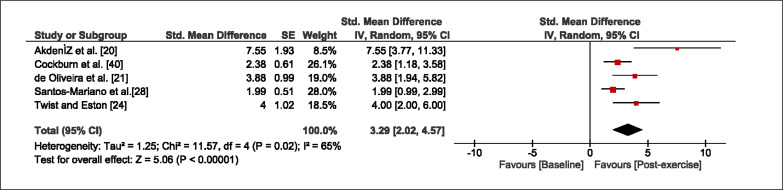
Forest plot for perceived muscle soreness at 72-hours after the muscle damaging protocol

**FIG. 6a:**
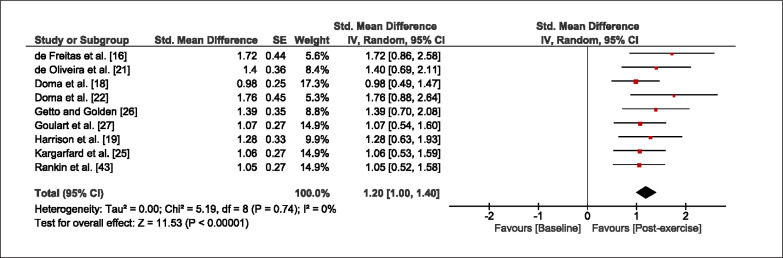
Forest plot for jump performance at 24-hours after the muscle damaging protocol

**FIG. 6b:**
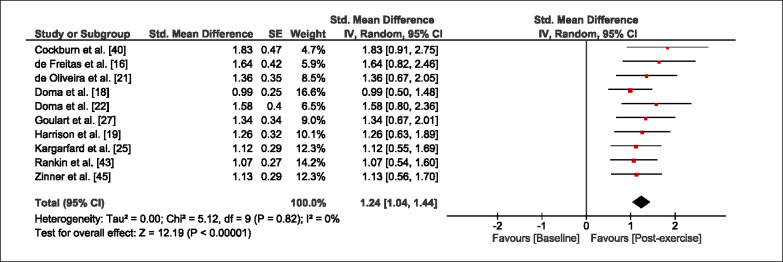
Forest plot for jump performance at 48-hours after the muscle damaging protocol

**FIG. 6c:**
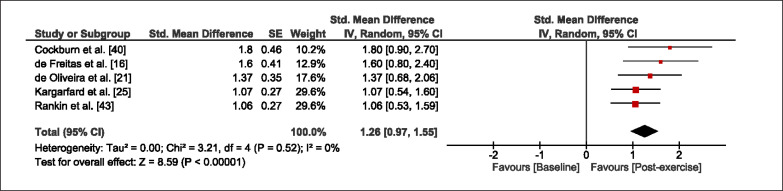
Forest plot for jump performance at 72-hours after the muscle damaging protocol

### Sensitivity Analysis

When studies that investigated resistance untrained participants were excluded, the primary outcome measures (Sprint p = ≤ 0.01; SMD = 1.46, 1.37, 1.27; COD p = ≤ 0.01; SMD = 1.30, 1.57, 1.18) and secondary outcome measures (CK p = ≤ 0.01; SMD = 3.47, 3.90, 3.60; DOMS p = ≤ 0.01; SMD = 3.08, 4.25, 3.91; CMJ p = ≤ 0.01; SMD = 1.30, 1.36, 1.32) did not significantly change the results across all respective timepoints. When studies utilising plyometric exercises were removed, no significant change was apparent amongst the primary and secondary outcome measures throughout 72-hours (Sprint p = ≤ 0.01; SMD = 1.48, 1.49, 1.46; COD p = ≤ 0.01; SMD = 1.18, 1.62, 1.02; CK p = ≤ 0.01; SMD = 2.89, 2.52, 3.03; DOMS p = ≤ 0.01; SMD = 2.34, 2.31, 2.15; CMJ p = ≤ 0.01; SMD = 1.19, 1.28, 1.38). Inversely, when studies utilising resistance exercises were removed, no significant change was apparent amongst the primary and secondary outcome measures across all timepoints (Sprint p = ≤ 0.01; SMD = 1.58, 1.57, 1.46; COD p = ≤ 0.01; SMD = 1.18, 1.62, 1.02; CK p = ≤ 0.01; SMD = 2.68, 2.84, 3.03; DOMS p = ≤ 0.01; SMD = 2.35, 2.47, 2.15; CMJ p = ≤ 0.01; SMD = 1.12, 1.24, 1.38). When studies that scored 25 or lower on the Kmet scores were excluded, the results were unaffected for all primary and secondary outcome measures and all timepoints (Sprint p = ≤ 0.01; SMD = 1.46, 1.46, 1.27; COD p = ≤ 0.01; SMD = 1.25, 1.42, 1.31; CK p = ≤ 0.01; SMD = 2.72, 2.65, 1.8; DOMS p = ≤ 0.01; SMD = 3.86, 4.03, 4.54; CMJ p = ≤ 0.01; SMD = 1.37, 1.46, 1.47). In some instances, authors did not report standard deviations for some outcome measures and as a result standard deviations were averaged. When such studies were excluded from the analysis, the significance of the results were unaffected for all primary and secondary outcome measures (Sprint p = ≤ 0.01; SMD = 1.44, 1.33, 1.18; COD p = ≤ 0.01; SMD = 1.25, 1.40, 1.18; CK p = ≤ 0.01; SMD = 2.72, 2.65, 1.8; DOMS p = ≤ 0.01; SMD = 3.86, 4.03, 4.54; CMJ p = ≤ 0.01; SMD = 1.25, 1.23, 1.27).

## DISCUSSION

The current systematic review and meta-analysis examined the acute effects of EIMD typically performed in a training context (i.e., resistance and/or plyometric exercises) on fundamental movement patterns, including sprints, COD, and CMJ performance. The inclusion criteria yielded 20 articles which: 1) utilised resistance or plyometric exercises; 2) compared post-exercise time points 24-hours onwards; 3) included sprint and COD performance. The meta-analysis revealed that, resistance training and plyometric exercises induced significant increase in muscle damage which impaired sprint and COD performance up to 72-hours post-exercise. In addition, EIMD was confirmed by changes in CK, DOMS, and CMJ for up to 72-hours post exercise.

Sprint and COD performances are impaired up to 72-hours following plyometric and resistance training. Our findings disagree with Silva et al [34] who found that sprint and COD performances recovered faster (48-hours) after an 11v11 football match. These athletes may have experienced lower match-induced stress given the physical demands are known to be inconsistent between playing positions, which may have reduced their pooled effect estimate of EIMD. For example, Bradley et al [46] found large positional differences in high intensity running in elite football players. Therefore, based on our findings, greater recovery may be required for more structured exercise sessions involving strenuous eccentric contractions (i.e., resistance training and plyometrics) when compared to team sport matches.

In the current meta-analysis, comparing RT and plyometric exercises showed a consistent acute impairment in sprint and COD performance, despite fundamental differences between the training modalities. Eccentric contractions are a significant component of plyometric and resistance exercise that are known to exacerbate the signs and symptoms of EIMD [47]. Several mechanisms may contribute to the symptom exacerbation such as increased mechanical stress, calcium homeostasis disruption and inflammation [48]. The mechanical stress sustained has shown to impair excitation-contraction coupling mechanism which in turn results in a later reduction of muscle strength [15]. Our meta-analysis supports these findings, demonstrating significant decreases in CMJ performance for up to 72-hours post-exercise. Sprinting and plyometric performance is performed optimally when morphological and neural components act in unison [49]. However, EIMD may disrupt these components (i.e., muscle architecture, tendon properties, motor unit recruitment and synchronicity) by increasing the displacement of the muscle fibres during stretch-shortening cycle movements causing slower running actions (e.g., acceleration) involved with the respective sprinting and COD tasks, as evidenced in this meta-analysis.

The current meta-analysis suggested that resistance and plyometric exercise impaired sprint and COD performance up to 72 hours post-exercise. However, when inspecting the papers individually, several studies reported non-significant differences at 24 [16, 17, 19, 26–29], 48 [16, 19, 21, 27–29, 42], and 72-hours [20, 21, 24, 25, 28, 29] post-exercise, which may be attributed to previous training experience of the participants involved in these studies. For example, the paper by Goulart et al [27] found no significant difference in 20 m sprint time over the 48-hour period in professional, resistance trained, female soccer players. Additionally, de Oliveira et al [21] found that COD performance did not change up to 72-hours post-exercise in under 19 male football players who participated in strength training 3 days per week. This trend may be explained by the repeated bout effect phenomenon, which is known to reduce EIMD symptomology via neural, mechanical, and cellular pathways with greater familiarity to eccentric contractions [50]. Thus, the detrimental effect of resistance or plyometric exercises has on sprint and COD performance appears to be attenuated in those who are previously exposed to strenuous exercises involving eccentric contractions (due to lower levels of EIMD).

Eccentric exercise is known to increase perception of DOMS and CK secretion. Both indirect muscle damage markers increase due to sarcomere trauma which activates the inflammatory response to repair intracellular damage [48]. Our meta-analysis showed that DOMS and CK were significantly increased at 24, 48, and 72 hours after resistance and plyometric exercise. These findings are in line with traditional research that has employed isokinetic contractions [14, 51, 52], suggesting similar soreness trends between monoarticular and multiarticular exercises. However, these findings should be considered with caution as receptor types and perception of soreness vary between individuals [53], resulting in high inter-study heterogeneity which is common among DOMS and CK values in meta-analyses [31–33]. Nonetheless, our findings confirm that resistance and plyometric exercises are muscle-damaging exercises, and athlete discomfort should be considered when planning subsequent training sessions (particularly those involving sprint and COD exercises).

According to the critical appraisal analyses, we found several methodological concerns from previous studies that should be addressed in future research. Firstly, whilst most studies reported key training variables for the muscle-damaging protocols, such as load, repetitions, sets, rest and exercise order, some studies did not provide sufficient methodological description to replicate the muscle-damaging protocol [24, 29, 40, 43–45]. Future research must clearly outline the muscle damaging protocols [30, 54, 55]. Secondly, limited number of studies provided replicable information on participants’ training history. This information is important as previous resistance training exposure can significantly reduce the level of muscle damage due to the repeated bout effect phenomenon [56]. Third, only few studies [17, 19–21] controlled for dietary and supplementation habits and implemented familiarisation sessions during the testing period. Future research should consider restricting foods known to reduce the signs and symptoms of EIMD [31–33] and incorporate familiarisation sessions to limit the learning effect. There are limitations that exist within the current systematic review and meta-analysis which should be noted. Firstly, the *I*^2^ values for CK and DOMS ranged between 65%–81%, indicating high inter-study heterogeneity. Secondly, articles not published in English were excluded, possibly biasing our meta-analysis from a cultural and linguistic standpoint. Thirdly, we combined studies with participants from various training backgrounds, which may have influenced the results due to the repeated bout effect. However, the sensitivity analyses indicated that removal of trained participants exhibited minimal impact on the overall meta-analyses. Fourth, whilst COD and sprint performance are representative of movements observed in sports, the findings from this meta-analysis may not translate to movement or sport-specific skills, such as throwing or kicking accuracy. Finally, whilst the inclusion criteria strictly included studies that utilised either plyometrics, resistance exercises or the combination as muscle-damaging protocols, the subtle differences in the type of exercises may have influenced our results. However, the sensitivity analyses showed that when studies with plyometric exercises were removed, the overall meta-analyses did not change.

## CONCLUSIONS

Our systematic review and meta-analysis demonstrated a significant impairment of sprint and COD performances for 24–72-hours following resistance and plyometric exercises. Therefore, coaches should monitor each athlete’s individual response to these training methods, adapting their exercise prescription and integrating appropriate time for recovery when EIMD is observed.
